# Lymphangioleiomyomatosis and pregnancy: a mini-review

**DOI:** 10.1007/s00404-024-07478-2

**Published:** 2024-04-09

**Authors:** Jieshu Zhou, Min Diao

**Affiliations:** 1 Department of Anesthesiology, West China Second University Hospital, Sichuan University, Chengdu, Sichuan China; 2 Key Laboratory of Birth Defects and Related Diseases of Women and Children, Ministry of Education, Sichuan University, Chengdu, Sichuan China

**Keywords:** Lymphangioleiomyomatosis, Pregnancy, Maternal health, Pulmonary function, mTOR

## Abstract

Lymphangioleiomyomatosis(LAM) is a slow progressive, rare cystic lung disease in women of reproductive age, associated with infiltration of the lung by atypical smooth muscle like cells, leading to the cystic destruction of the lung parenchyma. As LAM exclusively affects women of childbearing age, it can arise or exacerbate during pregnancy. Many patients with LAM are discouraged from pregnancy, although there is not much objective evidence effect on fertility. Patients diagnosed with LAM during pregnancy experience worse outcomes, so the safety of pregnancy is a vexing problem. What was worse, treatment strategies are limited on the effects of LAM on pregnancy outcomes. Pregnancy could be considered in LAM patients. Successful delivery in women with LAM depends on the condition of the LAM, which is in turn dependent on obstetricians and respiratory physicians. In this review, we describe the epidemiology, pathogenesis, diagnosis, clinical features and the treatment strategies of LAM during pregnancy.

## Background

Lymphangioleiomyomatosis (LAM) is a slow progressive, rare, multisystemic disease associated with cystic lung and abdominal tumors and chylous fluid due to abnormal proliferation and infiltration of smooth muscle cells, called LAM cells [1]. LAM cells are caused by mutations in the tuberous sclerosis complex genes (*TSC1/TSC2*) that result in activation of the mammalian target of rapamycin (mTOR), which regulates cellular growth and proliferation [2]. Progressive dyspnea and spontaneous pneumothorax are the most common clinical symptoms of LAM, especially in pregnant women [3]. Mounting evidence suggests that this disease exclusively affects women and exacerbates pregnancy and estrogen use [4, 5]. Notably, the European Respiratory Society (ERS) guidelines mention that pregnancy is associated with increased LAM complications [6]. Many patients with LAM avoid or are discouraged from pregnancy [7], although there is not much objective evidence. In this review, we describe the epidemiology, pathogenesis, diagnosis, and clinical features of LAM during pregnancy and highlight the treatment strategies. We aimed to explore the characteristics of LAM during pregnancy and provide evidence for a good outcome of pregnancy and maternal health.

## Epidemiology during pregnancy

LAM can occur sporadically (S-LAM) or accompany with tuberous sclerosis complex (TSC) (TSC-LAM). S-LAM is a rare disease restricted to premenopausal women. The prevalence of S-LAM was 4.9 per million women (range, 3.4–7.8 per million women) [8]. TSC-LAM has a high prevalence in women (range, 1–20 per million women), which increases with age [9]. It is about 30–40% TSC women has LAM [10]; however, TSC has no sex bias. Although TSC-LAM appears to be more common, mostly pregnant women with LAM for medical evaluation have sporadic LAM [11]. This paradox remains unexplained but may be ascribe to the lack of sex bias of TSC or because the prevalence of TSC-LAM is higher in older than in younger women. The average age at the diagnosis of S-LAM is 34 years [12]. In a retrospective study of pregnancy in patients with LAM, two-thirds of the patients were pregnant [13]. However, pregnancy is rare once the diagnosis is made. There are few firm data on the prevalence of LAM during pregnancy.

## Pathogenesis of LAM

Both TSC-LAM and S-LAM are caused by mutations in the tumor suppressor complex genes *TSC1* and *TSC2* which has been present in lung LAM cells [14]. TSC1 and TSC2 are negative regulators of mammalian target of rapamycin (mTOR), which regulates cellular growth and proliferation. Overactivation of the mTOR pathway in TSC-mutant cells results in LAM cell growth and proliferation [15]. Therefore, mTOR inhibition has been used as the main therapeutic strategy for LAM.

The source of LAM cells remains unknown; however, LAM lesions have been reported in the uterus [16, 17], and TSC2 gene mutations have been observed in the uterus and lungs of patients with S-LAM [18]. S-LAM has the stronger sex preference. Lung tissues from LAM patients were found to express high levels of both estrogen and progesterone receptors [19], which suggests the hormonal sensitivity of this disease. Estrogen may play a pivotal role in S-LAM, supported by the higher occurrence of the disease in women of reproductive age and the fact the complications of LAM in pregnancy are 11 times higher than at other times [20]. In vitro studies showed that estrogen induces growth of cultured TSC2-deficient cells and tumor cells derived from patients with LAM through pyruvate kinase M2 [21]. Gu et al. [22]. reported that estrogen-ERK2 pathway activated mTOR pathway by enhancing the effect of late response-gene *Fra1*. Oberstein et al. [23] also suggested that oral contraceptive pills, which contain high levels of estrogen, serve as catalysts to promote the occurrence of LAM. The female sex hormone environment has been implicated in the pathogenesis of LAM. Progesterone as another sex hormone is plays a key role in pregnancy maintenance. Progesterone was found to increase the migration and invasion of TSC2-deficient cells [24], and previous report also revealed that administration of progesterone with oophorectomy is the most effective therapeutic for LAM. However, the quality of evidence regarding hormone therapy is low.

## Presentation and clinical features during pregnancy

### Lung

The major pulmonary symptoms of LAM include dyspnea on exertion, pneumothorax, and chylothorax. Some patients have mild pulmonary symptoms 2–3 years before diagnosis, and the symptoms worsen during pregnancy [25]. Progressive dyspnea is usually the first symptom when LAM is diagnosed during pregnancy. Dyspnea is easily masked by other diagnoses such as chronic obstructive pulmonary disease and asthma; therefore, the diagnosis of LAM is often delayed [26]. Khaddour et al. [27]reported that a female with history of G3P2A1 presented with the chief complaint of worsening dyspnea, which was misdiagnosed as asthma during pregnancy. Progressive dyspnea may be caused by LAM cell destruction in the lung parenchyma and decreased lung function.

Spontaneous pneumothorax is a classic presentation of LAM with pregnancy. Over one-third of patients experience pneumothorax and about two-thirds experience recurrence of pneumothorax during pregnancy [26]. Chylothorax is a clinical manifestation in about 20% of LAM patients. The incidence of pneumothorax and chylothorax during pregnancy was 11 times higher than that at other times [20]. Pleurodesis is recommended for spontaneous pneumothorax, which can reduce the risk of pneumothorax recurrence [28]. However, chemical pleurodesis may cause maternal and fetal hepatic toxicity [29]. Thoracic drainage (intercostal drainage) is often used in patients with pneumothorax during pregnancy and is associated with reduced dyspnea [30]. In retrospective research shown that air travel increases the incidence of pneumothorax in patients with LAM, especially in those with larger cysts and lower lung function. The incidence of pneumothorax in LAM after air travel is approximately 1000 times higher than general women and is about threefold higher than baseline incidence [31]. However, current guidelines do not restrict patients with LAM from air travel [6]. We believe that the advice to avoid air travel should be adopted during pregnancy, because pregnant patients are at high risk of pneumothorax and reducing unnecessary air travel can reduce the incidence of pneumothorax and other lung complications.

### Extrapulmonary presentations

Extrapulmonary presentations, including renal angiomyolipomas (AMLs) and lymph node enlargement, are reported in some cases of pregnancy. Angiomyolipomas (AMLs) are benign tumors usually localized in the kidneys and occur in almost 90% of patients with TSC-LAMs and in approximately 30–40% of patients with S-LAM [26, 32]. AMLs usually present with abdominal pain, hypotension, and shock due to bleeding or rupture [33]. Rapid growth of AMLs has been reported in pregnant patients [34–36], which will lead to increase the rate of AMLs rupture. AMLs are atypical presentations of LAM; however, in some case reports of pregnancy complicated by LAM, the first symptoms experienced by most patients were abdominal pain and renal hemorrhage. The treatment for AMLs is arterial embolization, nephrectomy, cryoablation, and radiofrequency ablation. When tumors are less than 5 cm in size, cryoablation and radiofrequency ablation are the treatments of choice for most AMLs. When nephrectomy is not possible, embolization is considered the primary treatment. There is no confirmed effect of radiation on the baby if embolization is performed during pregnancy with AMLs (Table 1).

**Table 1:** Clinical features and treatment during pregnancy

Organ	Manifestation	Treatment
Lung	Dyspnea Pneumothorax Chylothorax	Oxygen therapy Pleurodesis Thoracic drainage
Renal	Abdominal pain Bleeding or rupture	Arterial embolization Nephrectomy Cryoablation Radiofrequency ablation

## Diagnosis and evaluation

### High-resolution computed tomography (HRCT)

Diffuse thin-walled cysts in the lung parenchyma are the most remarkable feature of LAM on high-resolution computed tomography scans. Although the characteristic cystic disease on CT alone has a sensitivity and specificity for the diagnosis of LAM is about 90%, it is considered insufficient to diagnose LAM [37]. Diagnosis of LAM requires exclusion of other causes of diffuse cystic lung diseases, such as chronic obstructive pulmonary disease, Langerhans cell histiocytosis, and cyst-forming metastatic lung tumors [25].

### VEGF-D and pulmonary function

Serum vascular endothelial growth factor-D (VEGF-D) testing has been suggested as a noninvasive method to confirm the diagnosis of LAM [6, 37, 38]. An elevated serum VEGF-D level >800 pg/mL has a sensitivity of 73% and a specificity of 100% and provides a definite diagnosis of LAM in patients with a clinical symptoms and characteristic chest high-resolution computed tomography result [6]. Serum VEGF-D testing reduces the need for invasive lung biopsy in patients with LAM, which can be used in the diagnosis of pregnancy complicated with LAM. Hirose et al. [39]. showed that the serum level of VEGF-D remains unchanged during pregnancy with LAM, suggesting that estrogen has little effect on serum VEGF-D levels. VEGF-D is able to predict the severity of the disease, and baseline serum VEGF-D levels are negatively correlated with pulmonary function testing [33, 40]. Both VEGF-D and pulmonary function testing can be used to assess disease progression. Pulmonary function tests mostly revealed an airflow obstructive impairment pattern or reduced pulmonary diffusion capacity in patients with LAM [41]. Pulmonary function testing of forced expiratory volume in one second (FEV1) has shown the strongest correlation with LAM, and the rate of decline in FEV1 has been variably reported as 75–135 mL per year in patients with LAM [42]. Pulmonary function testing is the most effective and non-invasive method for assessing the severity and progression of lung disease in LAM [43]. A lower baseline pulmonary function level is associated with high mortality in LAM. Most pregnancies in women with LAM are associated with acceleration of lung function decline [44]. Angelo et al. [45] performed a retrospective study of 16 LAM pregnant patients and showed that the percentage of predicted FEV1 and diffusion in the lung of carbon monoxide (DLCO) were lower than those before pregnancy. Therefore, we concluded that pregnant LAM patients have poorer Pulmonary function with lower FEV1, forced vital capacity (FVC), and DLCO. Time-course monitoring of pulmonary function during pregnancy is vital to assessing LAM progression.

### Biopsy

Biopsy is the gold standard for diagnosing LAM. The lung biopsy can be performed through transbronchial or video-assisted thoracic surgery when the diagnosis cannot be made noninvasively in LAM patients [46].

## Treatment during pregnancy

### mTOR inhibitors

The American Thoracic Society and Japanese Respiratory Society Guidelines recommend sirolimus as a first line treatment for LAM because it improves lung function and reduces serum VEGF-D levels by inhibiting the mTOR pathway. The Multicenter International LAM Efficacy of Sirolimus (MILES) trial showed that sirolimus treatment improves FVC and FEV1 in patients with LAM and moderate lung impairment [47]. With respect to effective dosing, sirolimus was administered at 2 mg/d, and the blood concentration was maintained at 5–15 µg/L. Regrettably, sirolimus is a risk category C for pregnancy, and guidelines advise discontinuing it 12 weeks before pregnancy. Sirolimus is also an effective treatment for LAM while the therapy continues, but disease progression resumes once treatment is discontinued [25]. Sirolimus has not been used in pregnant LAM patients, except in some case reports. Shan et al. [11] reported that sirolimus increased the risk of spontaneous abortion and premature delivery during pregnancy. In some unintended pregnancy cases and retrospective data, the use of the drug before or during pregnancy did not increase adverse neonatal outcomes (Table 2), and the use of small doses (1–2 mg/d) can stabilize lung function in pregnant women. Faehling et al. [48, 49] reported the case of a patient with long-term LAM administered sirolimus who had a second successful pregnancy. They believe that low blood levels of sirolimus (3–5 µg/L) may be safe in pregnant LAM patients, which is in line with previous reports on treatment with low-dose sirolimus (< 5 µg /mL) being effective in stabilizing lung function. However, there is little evidence of sirolimus use in pregnancy with LAM. The safety and efficacy of sirolimus in pregnancy requires robust research data in the long term.

**Table 2:** Patients with LAM and a history of pregnancy after taking sirolimus

patient	Type of LAM	Age of diagnosis(years)	Duration of taking sirolimus (month)	Dose (mg)	Serum level（ug/L）	Time of discontinuation（weeks）	Age of pregnancy(years)	Mode of delivery	Gestational weeks	Complication of LAM	Obstetric diseases	infants
1	S-LAM	29	53	1.5	7.8	At pregnancy 8weeks	34	CS	37	None	None	Healthy
2	S-LAM	30	46	1.5	7	At pregnancy 5weeks	34	CS	35	Rupture of AMLs	None	Healthy
3	S-LAM	25	70	1	NA	At pregnancy 4weeks	35	CS	38	None	None	Healthy
4	S-LAM	34	29	1	6	Before pregnancy 27weeks	37	VD	39	None	None	Healthy
5	S-LAM	26	12	1	NA	Before pregnancy 8weeks	27	VD	38	PX	None	Healthy
6	S-LAM	30	6	1	NA	Before pregnancy 12weeks	33	CS	37	PX	None	Healthy
9	S-LAM	23	30	NA	NA	At pregnancy	39.8	CS	40	None	None	Healthy
10	NA	25	9	2	2	At pregnancy 7–23 weeks	29	CS	32	PX	None	Healthy
11	NA	25	61	2	03-May	no	34	CS	32	None	None	Healthy

*S-LAM* sporadic lymphangioleiomyomatosis, *TSC* tuberous sclerosis complex, *AMLs* angiomyolipomas, *PX* pneumothorax, *CS* cesarean section, *VD* vaginal delivery

Recent studies on mTOR signaling have suggested the use of other drugs, such as biguanides [50], statins [51], and doxycycline [52]. Given the favorable safety features of biguanide in pregnancy, it might be a good alternative treatment option for pregnant LAM patients [53, 54]. An ongoing clinical trial investigating the safety and efficacy of statins and mTOR inhibitors in patients with LAM has completed enrolment; however, the safety of statins during pregnancy remains questionable. Matrix metalloproteinases (MMPs) may contribute to cyst formation in patients with LAM. Treatment with doxycycline, an inhibitor of MMPs, has been investigated in this regard. The case report suggests a potential benefit of doxycycline therapy for patients with LAM [24], and lung tissues obtained from patients with LAM were found to express high concentrations of MMPS-2 and MMPS-9 [55]. Doxycycline is effective for the treatment of LAM [56], but the use of doxycycline during pregnancy has historically been discouraged due to potential adverse effects on the fetal teeth during 16 weeks postconception. Although a study assessed doxycycline use during pregnancy [57], the data are insufficient to state that there is no risk. So clinical trial guidelines do not suggest the use of doxycycline as a treatment for LAM because there are no beneficial effects in LAM patients who already have lung function impairment. The use of doxycycline for treatment of LAM during pregnancy has not been reported yet.

### Hormone treatment

Pregnancy and LAM are inseparable. Although hormone-related treatments, such as oophorectomy and estrogen, progesterone, and GnRH treatments, may have potential against LAM, pregnancy does not allow hormonal manipulation using progesterone, estrogen antagonists, or luteinizing hormone-releasing hormone agonists. There is a lack of robust clinical randomized double-blinded placebo-controlled trial studies on the management of LAM in pregnancy using sex hormonal agents. Oprescu et al. [58] showed that hormonal treatment was linked to an increased risk of death in patients with LAM. Therefore, therapeutic strategies using female sex hormones have not been successful and are, therefore, not recommended by the guidelines.

### Other pharmacological agents

Reversible airway obstruction occurred in 20% of patients with LAM and association with prognosis [59]. Bronchodilator drugs are used in patients with asthma-like symptoms caused by LAM. Activation of protein kinase A(PKA) by treatment with β-agonists increased S6 phosphorylation level independent of mTOR [60]. Retrospective data from LAM patients showed that a combination of bronchodilators with sirolimus is better than sirolimus alone for stabilization of pulmonary function.

To date, there are only few effective pharmacological treatments for pregnant LAM patients, and the benefits of available medications during pregnancy are debatable owing to side effects on the fetus. Treatment of LAM in pregnancy, therefore, focuses on symptom management and prevention of complications.

### Lung transplantation

Lung transplantation is the last treatment strategy for patients with worsened lung function at the end stage. Lung transplantation is an effective treatment that could improve the quality of life of patients with LAM [61, 62]. In a 2021 analysis, Ji Zhang et al. [63]evaluated 25 patients with LAM who underwent lung transplantation in China between 2010 and 2018 and found that 19 (76%) patients had a history of pregnancy and six (24%) patients had a recent history of pregnancy. However, this article does not show relationship between lung transplantation and pregnancy.

## Effect of pregnancy on LAM

Pregnancy is an important issue for patients diagnosed with LAM. In a registry study of 230 patients with LAM, two-thirds of the patients had a history of pregnancy. According to Cohen et al.[13], 78.8% of the patients had favorable pregnancy outcomes before LAM diagnosis. LAM diagnosis during pregnancy was related to a higher incidence of complications, such as recurrent pneumothorax [20], rupture of renal AMLs [64], and loss of pulmonary function [45].

## Effect of LAM on pregnancy

Patients with LAM have more premature births and miscarriages than those without LAM. In line with these reports, miscarriage was associated with less fertile LAM patients [65]. In terms of delivery methods, a retrospective study showed that 75% (12/16) of the patients underwent cesarean section, and only 25% of the patients gave birth naturally. A high rate of cesarean sections was observed owing to the reduced risk of rupture of AMLs and pneumothorax during labor. The timing of termination of pregnancy is mainly based on the condition of the diseases progression. Pregnancy may adversely affect the prognosis of patients with LAM; however, it was not associated with their survival.

## Conclusion

Pregnancy is a vexing problem for reproduction women owing to reduced fertility, increased risk of miscarriage, premature delivery, and complications of LAM. Successful delivery in women with LAM depends on the condition of the LAM, which is in turn dependent on obstetricians and respiratory physicians. Pregnancy could be considered in LAM patients, pregnancy plans should be made according to individual conditions, and pulmonary function should be monitored in real time during pregnancy. Future research should explore strategies to avoid complications and improve pulmonary function, such as use of drugs in pregnant women and physical exercise.

## Abbreviations

LAM: Lymphangioleiomyomatosis
mTOR: Mammalian target of rapamycin
ERS: European Respiratory Society
S-LAM: Sporadic lymphangioleiomyomatosis
TSC-LAM: Tuberous sclerosis complex lymphangioleiomyomatosis
TSC: Tuberous sclerosis complex
AMLs: Angiomyolipomas
VEGF-D: Vascular endothelial growth factor-D
GnRH: Gonadotrophin-releasing hormone
FEV1: Forced expiratory volume in one second
DLCO: Diffusion in the lung of carbon monoxide
FVC: Forced vital capacity
MMPs: Matrix metalloproteinases
